# Cannabis and psychosis: from the population attributable fraction to the importance of gender differences

**DOI:** 10.1192/j.eurpsy.2025.45

**Published:** 2025-08-26

**Authors:** C. Hjorthøj

**Affiliations:** 1 Copenhagen Research Center for Mental Health - CORE; 2 Department of Public Health, University of Copenhagen, Copenhagen, Denmark

## Abstract

**Abstract:**

I will present data from a range of Danish studies on both cannabis-induced psychosis and Danish epidemiological studies on the association between cannabis and psychotic disorders such as schizophrenia. A deeper understanding of these associations is important. If the consistent association that we have observed for many decades is indeed causal, then cannabis is perhaps the single most important preventable risk factor for schizophrenia that we have identified to date. The development over time of the population attributable risk fraction, gender differences, and other novel areas will be covered in the presentation.

**Disclosure of Interest:**

None Declared

